# Effects of lidocaine administration via the perforated outer cuff of a dual-cuff endotracheal tube and remifentanil administration on recovery from general anaesthesia for female patients undergoing thyroidectomy: a single centre, double-blind, randomised study

**DOI:** 10.1186/s12871-022-01734-1

**Published:** 2022-06-22

**Authors:** Ping Li, Dewei Li, Linan Wang, Fei Ye, Bo Yang, Lina Yu, Sujuan Fang, Zhilan Tong, Qing Yin, Yongyong Shi, Xiangyu Li, Gaofeng Zhao

**Affiliations:** Department of Anesthesiology, The Second Affiliated Hospital of Guangzhou University of Chinese Medicine: Guangdong Provincial Hospital of Chinese Medicine, Guangzhou City, Guangdong Province 510120 People’s Republic of China

**Keywords:** Anaesthesia, General, Anaesthesia topical, Lidocaine, Endotracheal tube, Modified dual-cuff, Opioid, Remifentanil

## Abstract

**Background:**

Cough caused by endotracheal tube (ETT) placement is ubiquitous and correlates with adverse outcomes. Remifentanil administration via target-controlled infusion (TCI) is one of the cough prevention measures used during recovery. In a pilot study, lidocaine administered via the perforated outer cuff of a dual-cuff endotracheal tube was also found to prevent cough due to ETT placement. We therefore compared these two cough prevention approaches during recovery after thyroidectomy in a single-centre, double-blind, randomised study conducted in China during the period from 09/10/2020 to 30/04/2021.

**Methods:**

Ninety-eight female patients aged 18–65 years with American Society of Anaesthesiologists Physical Status scores of I and II were scheduled to undergo thyroidectomy. The ETT contained an internal cuff covered by a perforated outer cuff to allow for lidocaine delivery. Patients were randomised to receive either 4 ml of saline solution (Group R, *n* = 49) or 4 ml of 2% lidocaine in the outer cuff (Group L, *n* = 49) at the beginning of skin suturing. Remifentanil (2 ng/ml) was maintained in Group R until extubation, while remifentanil was maintained in Group L until the end of skin suturing. The primary outcome was cough during patient transfer, at 1 min before extubation, and at extubation. The secondary outcomes were haemodynamics and other recovery parameters.

**Results:**

Primary outcomes were compared between remifentanil *vs.* lidocaine application, namely, the incidence of cough during patient transfer (0% in Group R *vs.* 0% in Group L), at 1 min before extubation (22.45% in Group R *vs.* 4.08% in Group L; *P* = 0.015), and at extubation (61.22% in Group R *vs.* 20.41% in Group L; *P* < 0.001). Compared with remifentanil, lidocaine more effectively decreased heart rate elevation and hypoxemia at 5 min after extubation, the spontaneous respiration recovery time, the extubation time, the duration of post-anaesthesia care unit (PACU) stay, Richmond Agitation-Sedation Scale scores in the agitated range and Critical-Care Pain Observation Tool scores.

**Conclusion:**

Lidocaine administered via the perforated outer cuff of the ETT significantly improved recovery from general anaesthesia compared to remifentanil in female patients after thyroidectomy.

**Trial registration:**

Chinese Clinical Trial Registry (No. ChiCTR2000038653), registered on 27/09/2020.

**Supplementary Information:**

The online version contains supplementary material available at 10.1186/s12871-022-01734-1.

## Introduction

Cough is ubiquitous (76–80%) during recovery from general anaesthesia and endotracheal tube placement [1, 2], which correlates with hypertension and tachycardia [3, 4], agitation [5] and postoperative haemorrhage [6]. Cough prevention during recovery is recommended for patients with suspected or confirmed coronavirus disease (COVID-19), as it can reduce airborne particles and droplets [7]. Cough prevention is also imperative for reducing the incidence of breathing difficulty associated with haemorrhage after thyroidectomy [8].

Remifentanil administration via target-controlled infusion (TCI) is one of the cough prevention measures used during recovery [9, 10]. In addition, to adequately reduce the stimulation of the tracheal mucosa in contact with the cuff of the EET, we modified a dual-cuff, creating a “disposable reinforced tracheal intubation double balloon dosing-type” endotracheal tube (Supplemental Fig. 1, Xi’an Shen Lan Biomedical Engineering Co., Ltd, China) (Supplemental Video 1), and found that the topical administration of lidocaine via the dual-cuff ETT could effectively prevent cough during recovery. In the pilot study, one patient was intubated with the dual-cuff ETT while sitting up in bed and signing her name (Supplemental Video 2). This dual-cuff ETT (Supplemental Fig. 2B) contains an internal cuff covered by an outer cuff. The small-bore channel is incorporated within the wall of the endotracheal tube and is the channel that delivers lidocaine to the perforated outer cuff through 16 small holes (Supplemental Video 3). The benefit of a modified dual-cuff ETT is that the tracheal mucosa in contact with the outer cuff receives topical anaesthesia by lidocaine via the perforated outer cuff without deflation of the internal cuff, so it is not restricted by the timing of lidocaine administration.

**Fig. 1:**
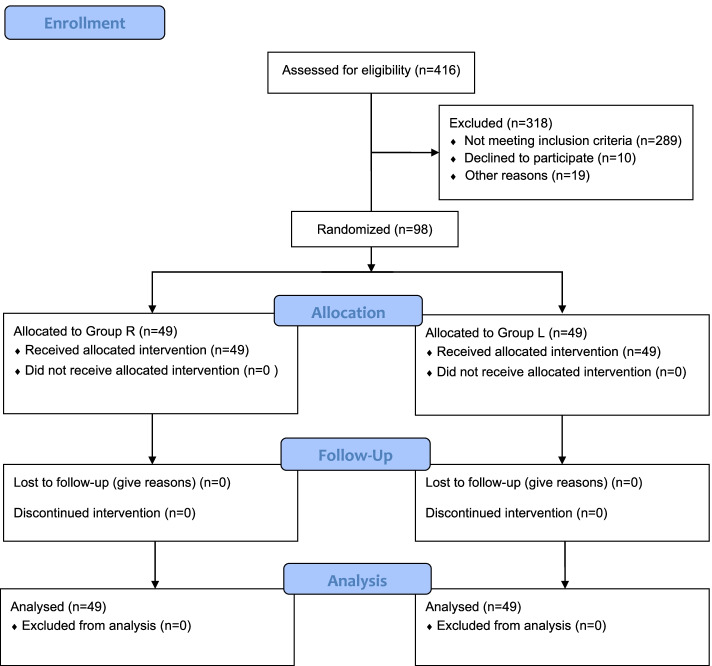
CONSORT diagram of the randomised trial. R, remifentanil; L, topical lidocaine via the perforated outer cuff

**Fig. 2:**
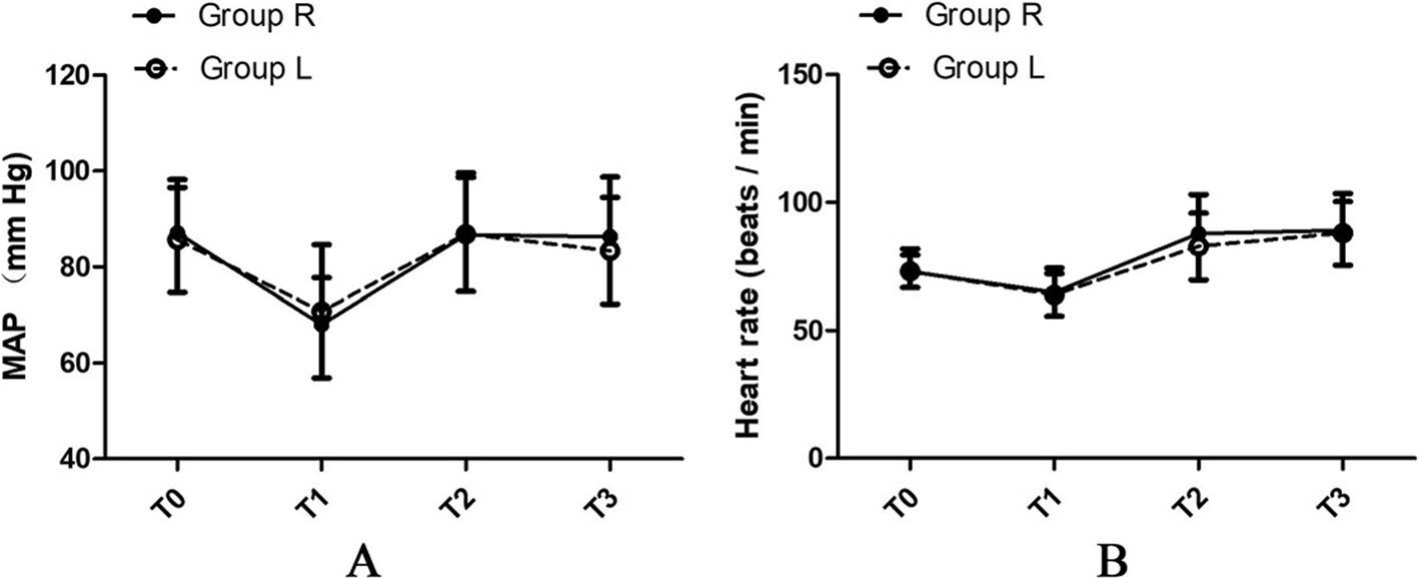
Haemodynamic changes during emergence from general anaesthesia. Data are expressed as the mean (standard deviation). MAP is shown in panel **A** and heart rate is shown in panel **B** R, remifentanil; L, topical lidocaine via the perforated outer cuff; MAP, mean arterial pressure; T0, one day before surgery; T1, the end of surgery; T2, 1 min before extubation; T3, 5 min after extubation

This single-centre, double-blind, randomised study was designed to evaluate the hypothesis that cough prevention with the topical lidocaine approach via the dual-cuff ETT before extubation would be better than that with remifentanil administration during recovery after thyroidectomy. Cough during recovery was chosen as the primary outcome for further study of the potential benefits of a modified dual-cuff ETT, such as the prevention of cough during the recovery of patients with suspected or confirmed COVID-19. The main secondary outcomes included haemodynamics, the time to recovery, hypoxemia, and other recovery parameters.

## Materials and methods

### Ethics and registration

Ethical approval for this study (Ethical Committee IRB: ZF2020-130.2–01) was provided by the Ethical Committee of Guangdong Provincial Hospital of Chinese Medicine, Guangzhou City, Guangdong Province, People’s Republic of China (Chairperson Prof. Jun Liu) on 28/08/2020. Written informed consent was obtained from each patient before study participation. The full trial protocol was registered before patient enrolment in the Chinese Clinical Trial Registry (ref: ChiCTR2000038653) on 27/09/2020 and adheres to the Consolidated Standards of Reporting Trials guidelines [11] and the Declaration of Helsinki.

### In vitro experiments

To achieve an even distribution of lidocaine around the cuff, we modified a dual-cuff endotracheal tube and conducted a dyeing experiment in vitro. We evenly punctured small holes through the outer cuff as a dye liquid delivery channel. As the tracheal diameter ranges from 8.9–17 mm in women [12], a tube with an internal diameter of 14 mm was used to simulate the trachea, and graph paper was placed on the internal surface of the tube to record the outcome of the experiment. The dual-cuff ETT was placed into a 14 mm tube lined with graph paper, and the internal cuff pressure was maintained at 25 cmH_2_O. To optimise the number of holes and the volume of dye liquid, we assessed the difference between 8 and 16 holes by using 1–4 ml of dye liquid to simulate lidocaine; this experiment was performed in triplicate. The dye liquid was fully transferred from the outer cuff by injecting 20 ml of air into the outer cuff.

Supplemental Fig. 2A shows the results of the dyeing experiment. There was no interaction between the number of holes (8 and 16 holes) and the dye liquid volume (1, 2, 3, and 4 ml) (*P* = 0.064). Pairwise comparisons of the area showed significant differences based on the number of holes (796.67 mm^2^ for 16 holes *vs.* 584.17 mm^2^ for 8 holes,* P* = 0.021) and the volume (937.67 mm^2^ for 4 ml *vs.* 477.50 mm^2^ for 1 ml,* P* < 0.001; 937.67 mm^2^ for 4 ml *vs.* 634.83 mm^2^ for 2 ml, *P* = 0.010; and 937.67 mm^2^ for 4 ml *vs.* 711.67 mm^2^ for 3 ml,* P* = 0.033). Based on these results, 16 holes and 4 ml of dye liquid was determined to be the optimal combination. This combination was subsequently used in vivo.

### In vivo experiment

#### Study design

This single-centre, double-blind, randomised study was conducted at the Department of Anesthesiology, Guangdong Provincial Hospital of Chinese Medicine in Guangzhou, China, from 09/10/2020 to 30/04/2021.

#### Patients

All 98 females with ASA Physical Status scores of I–II, aged 18–65 years, a height of 140–180 cm and weight of 40–85 kg planned to undergo elective thyroidectomy due to a unilateral thyroid neoplasm (T1/T2 N0 M0 in Tumor-Node-Metastasis System/American Joint Committee on Cancer). The first patient was enrolled on 14/10/2020. The following patients were excluded: patients using a nerve integrity monitor (NIM) standard reinforced electromyography (EMG) endotracheal tube; patients with signs of a difficult airway, perioperative aspiration, or psychosis; patients with an acute respiratory tract infection lasting less than 2 weeks; patients who smoked less than 48 h before the procedure; patients who had an allergic reaction to lidocaine or a beta-adrenergic receptor line; and patients with serious cardiovascular, pulmonary, hepatic, or renal disease.

#### Randomisation

Patients were randomly allocated at a 1:1 ratio to receive either 4 ml of 2% lidocaine in the outer cuff (Group L) or the predicted effect-site concentration (Ce) of 2.0 ng/ml of TCI remifentanil (Group R), according to a computer-generated centralised random table with no block size and stratification factors. A unique randomisation number was removed from the sequentially numbered containers on the morning of surgery. The first anaesthetist generated the random allocation sequence, performed the randomisation, enrolled the participants, and assigned the participants to interventions, but he was not at all involved in anaesthesia management.

#### Blind evaluation

Three anaesthetists and 3 anaesthetist nurses participated in this study to conduct a blinded evaluation. The first anaesthetist was the only one with knowledge of the group assignments. The other anaesthetists, anaesthetist nurses, and patients were blinded to the groups. The first anaesthetist completed the following tasks at the beginning of skin suturing: shielding the TCI pump from other anaesthetists, controlling the TCI pump, and providing 4 ml of 2% lidocaine or 4 ml of saline solution to the second anaesthetist, who was blinded to the actual treatments. The second anaesthetist performed anaesthesia management, including intraoperative and postoperative care, except for the control of the TCI pump at the beginning of skin suturing. The third anaesthetist and 3 anaesthetist nurses recorded data for the variables in the post-anaesthesia care unit (PACU).

#### Intraoperative and postoperative care

Basic monitoring was performed at 3 min intervals. All patients received induction medication, including 1.5 mg/kg propofol, 0.2 mg/kg cisatracurium, the predicted effect-site concentration of TCI remifentanil (Ce 3.5 ng/ml) and 0.4 µg/kg sufentanil to facilitate tracheal intubation. Remifentanil was administered using a TCI pump (targeted effect-site TCI, Minto model and CONCERT-III, Guangxi VERYARK Technology Co., Ltd, China). All patients received a 7.0 mm modified dual-cuff ETT, and the internal cuff pressure was maintained at 20–30 cmH_2_O with a hand pressure gauge (Hi-Lo™ Hand Pressure Gauge, VBM Medizintechnik GmbH, Germany). Sevoflurane (1.2–2.5%) and TCI remifentanil (Ce 2–5 ng/ml) were used to maintain the mean arterial pressure (MAP) and heart rate (HR) within 20% of baseline (values at one day before surgery). The nasopharyngeal temperature was maintained at 36–37 ℃. The tidal volume and ventilatory frequency were adjusted using end-tidal carbon dioxide (ETCO_2_) at 34–45 mmHg.

At the beginning of skin suturing, sevoflurane was maintained at 1.4–1.5 times the end-tidal concentration, and remifentanil was maintained at 2 ng/ml. At the same time, 0.5 mg/kg of ketorolac was administered to relieve pain, and 4.48 mg of tropisetron was administered to prevent nausea and vomiting. Neostigmine (50 µg/kg) and atropine (15 µg/kg) were administered to reverse the neuromuscular block to confirm a train-of-four response greater than 90%. Patients with an ETT were randomised to receive either 4 ml of 2% lidocaine in the outer cuff (Group L) or 4 ml of saline solution (placebo) in the outer cuff (Group R), followed by 20 ml of air in the outer cuff at the beginning of skin suturing (each time the internal cuff pressure was less than 30 cmH_2_O). Remifentanil was maintained at 2 ng/ml in Group R and turned off at the time of tracheal extubation. Remifentanil in Group L and sevoflurane in both groups were turned off at the end of skin suturing (the end of surgery).

Patients with an ETT were transferred to the post-anaesthesia care unit (PACU) after confirmation of stable haemodynamics. During the transfer of patients (the phase from the operating table to the PACU), 2.0 ng/ml remifentanil by TCI was maintained for Group R, whereas remifentanil was stopped for Group L. The phase from the end of surgery to the beginning of transfer took 4 min, and patient transfer took 2 min. All the patients were taken to the PACU with the shielded TCI pump. Recovery profiles were recorded in the PACU by video after obtaining the patients’ consent. Manual ventilation was provided until the patient breathed spontaneously. Mild hypercapnia (end-tidal carbon dioxide, ETCO_2_ of 45–55 mmHg) was permitted to promote spontaneous respiration. Continuous verbal stimuli were used to prompt the patients to open their eyes without any other stimuli. After opening their eyes, the patients were asked to breathe deeply and nod their head. Extubation was completed when the patients could take an adequate breath on command (ETCO_2_ < 50 mmHg and ventilatory frequency > 12 beats/min), nod and shake hands on command and achieve spontaneous deglutition.

#### Primary outcome

The primary outcome was the incidence of cough during the recovery period. A cough was defined as any evidence of irritation from the tracheal tube [13]. Cough was recorded during patient transfer (the phase from the operating table to the PACU), at 1 min before extubation and at extubation.

#### Secondary outcomes

Data from the following five time points were analysed: T0, one day before surgery (baseline); T1, the end of surgery; T2, 1 min before extubation; T3, 5 min after extubation; and T4, 20 min after extubation. The secondary outcomes included the following variables: MAP and HR at the above five time points; elevation of blood pressure and heart rate at T2, T3 and T4 (MAP and HR increase by 30% from their respective values at baseline); eye opening time (time period from the end of surgery to first eye opening on command); consciousness recovery time (time period from the end of surgery to nodding on command); spontaneous respiration recovery time (time period from the end of surgery to an ETCO_2_ < 55 mmHg and a ventilatory frequency > 10 beats/min); extubation time (time period from the end of surgery to extubation); duration of PACU stay (the time period from the end of surgery to leaving the PACU); the Richmond Agitation-Sedation Scale (RASS) score [14] at T2; and the Critical-Care Pain Observation Tool (CPOT) score [15] at T2.

In addition, the secondary outcomes were a composite of the following variables: hypoxemia at T3 and T4 (less than 95% of Spo_2_); the sedation grading system (SGS) [16] at T3; postoperative pain at T4 (more than 5 points on the visual analogue scale); residual sedation at T4 (less than a Grade 2 on the SGS); nausea and vomiting at T4 (need for drugs); and pharyngalgia at T4 (swallowing with more than 3 points on the visual analogue scale).

#### Sample size calculation

PASS^11^ statistical software (NCSS, LLC) was used to calculate the sample size. The incidence rate of cough during recovery from general anaesthesia was 76% [1]. Based on the assumption that Ce 2.0 ng/ml of TCI remifentanil could suppress cough by 90% [17] and 4 ml of 2% lidocaine used with the dual-cuff endotracheal tube could suppress cough by 50% [16, 18], the proportion of cough suppression was 68.4% in the TCI remifentanil group and 38% in the lidocaine group. Forty-eight patients in each group would be required for 80% power and α = 0.05 with a two-sided Fisher exact test.

#### Statistical analysis

All values are expressed as the mean ± standard deviation (SD), median (25% – 75%) or number (proportion). Continuous variables with a normal distribution were assessed with *a t test* or repeated measures analysis of variance with Bonferroni correction. Continuous variables with nonnormal distributions were assessed using the Mann–Whitney U test. Categorical data were assessed using the χ2 test or Fisher’s exact test. SPSS Statistics 20 (SPSS Inc., Chicago, IL, USA) was used to assess the data. Statistical significance was set at *P* < 0.05.

## Results

### In vivo experiments

#### Patients

We assessed 416 patients, 98 of whom were enrolled in this study. All 98 patients completed the study [Group L (*n* = 49); Group R (*n* = 49)] (Fig. 1). The patient characteristics were similar between the two groups (Table 1).

**Table 1:** Characteristics of the Patients Randomised between the Two Groups

Characteristic	Group R (*n* = 49)	Group L (*n* = 49)	Z/Mean Difference/OR (95% CI)	*P*
Age (years), median (25% – 75%)	49 (37–57)	39 (33–50.5)	-1.94 (0.05–0.06)	0.052^a^
ASA physical status (I/II), no. (%)	41(84%)/8(16%)	40(82%)/9(18%)	1.153 (0.405–3.286)	1^b^
Height (cm), mean ± SD	158.2 ± 5.07	158.9 ± 5.23	-0.70 (-2.72–1.41)	0.532^c^
Weight (kg), median (25% – 75%)	55 (50–63.5)	55 (49.5–60)	-0.63 (0.52–0.54)	0.531^a^
Duration of surgery (min), median (25% – 75%)	84 (68–100.5)	80 (71–99.5)	-0.44 (0.65–0.67)	0.659^a^
Duration of anaesthesia (min), median (25% – 75%)	121 (109–141)	120 (108.5–132.5)	-0.47 (0.64–0.66)	0.642^a^

*Abbreviations*: *ASA* American Society of Anesthesiologists, *R* TCI of remifentanil, *L* Topical lidocaine via the perforated outer cuff, *CI* Confidence interval, *OR* Odds ratio, *SD* Standard deviation

^a^ Mann–Whitney U test

^b^ Fisher’s exact test

^c^ *t test*

#### Primary outcomes

There were no patients with cough during patient transfer. The incidence of cough during T2 was significantly higher in Group R than in Group L (22.45% in Group R *vs.* 4.08% in Group L; *P* = 0.015). The incidence of cough during extubation was also significantly higher in Group R than in Group L (61.22% in Group R *vs.* 20.41% in Group L; *P* < 0.001) (Table 2).

**Table 2:** Primary Outcome and Related Parameters

Time	Cough	Group R (*n* = 49)	Group L (*n* = 49)	OR (95% CI)	*P*
Patient transfer	Cough occurrence, no. (%)	0 (0%)	0 (0%)		
1 min before tracheal extubation	Cough occurrence, no. (%)	11 (22.45%)	2 (4.08%)	0.15 (0.03–0.70)	0.015^a^
Tracheal extubation	Cough occurrence, no. (%)	30 (61.22%)	10 (20.41%)	0.16 (0.07 – 0.40)	< 0.001^a^

*Abbreviations: R* TCI of remifentanil, *L* Topical lidocaine via the perforated outer cuff, *CI* Confidence interval, *OR* Odds ratio. Transferring patients, the phase from the operating table to the post-anaesthesia care unit

^a^ Fisher’s exact test

#### Secondary outcomes

The secondary outcomes are shown in Table 3. The groups did not differ significantly in terms of MAP or HR (Fig. 2), and the incidence of elevated blood pressure was similar between the groups. The occurrence of heart rate elevation at T2 and T4 was also similar between the groups, but the heart rate elevation at T3 (*P* = 0.0028) was significantly lower in Group L than in Group R. The time related to recovery from remifentanil *vs.* lidocaine was as follows: eye opening time, 8.3 min in Group R *vs.* 7.7 min in Group L (*P* = 0.460); consciousness recovery time, 9.2 min in Group R *vs.* 8.1 min in Group L (*P* = 0.346); spontaneous respiration recovery time, 12.3 min in Group R *vs.* 8.9 min in Group L (*P* < 0.001); extubation time, 15.0 min in Group R *vs.* 11.0 min in Group L (*P* = 0.009); and duration of PACU stay, 55.4 min in Group R *vs.* 46.4 min in Group L (*P* = 0.001). The RASS scores in the agitated range were significantly lower in Group L than in Group R (*P* = 0.012), but there was no significant difference in scores in terms of the alert, calm, and sedation ranges. CPOT scores were significantly higher in Group R than in Group L (*P* = 0.003). The occurrence of hypoxemia was significantly lower in Group L than in Group R at T3 (*P* < 0.001), but there was no significant difference observed at T4. The groups did not differ significantly in grade 2/grade 3 sedation at T3, postoperative moderate pain, nausea and vomiting, or pharyngalgia at T4. No residual sedation was observed at T4 in either group.

**Table 3:** Secondary Outcomes and Related Components

Components	Group R (*n* = 49)	Group L (*n* = 49)	Z/Mean Difference/OR (95% CI)	*P*
Elevation of blood pressure at T2, no. (%)	3 (6.12%)	3 (6.12%)	1.00 (0.19–5.22)	1^b^
Elevation of blood pressure at T3, no. (%)	2 (4.08%)	1 (2.04%)	2.04 (0.18–23.29)	1^b^
Elevation of blood pressure at T4, no. (%)	2 (4.08%)	2 (4.08%)	1.00 (0.14–7.40)	1^b^
Elevation of heart rate at T2, no. (%)	13 (26.53%)	5 (10.20%)	3.18 (1.04–9.75)	0.066^b^
Elevation of heart rate at T3, no. (%)	16 (32.65%)	6 (12.25%)	3.48 (1.23–9.85)	0.028^b^
Elevation of heart rate at T4, no. (%)	6 (12.25%)	7 (14.29%)	0.84 (0.26–2.70)	1^b^
Time related to recovery				
Eye opening time (min), mean ± SD	8.3 ± 4.22	7.7 ± 3.05	0.6 (-0.93–2.03)	0.460^c^
Consciousness recovery time (min), median (25% – 75%)	9.2 (6.07–11.79)	8.1 (6.12–10.56)	-0.94 (0.35–0.37)	0.346^a^
Spontaneous respiration recovery time (min), median (25% – 75%)	12.3 (9.07–21.00)	8.9 (7.54–11.25)	-3.72 (0.00–0.0005)	< 0.001^a^
Extubation time (min), median (25% – 75%)	15.0 (11.00–22.00)	11.0 (10.00–15.00)	-2.60 (0.007–0.011)	0.009^a^
Duration of PACU stay (min), mean ± SD	55.4 ± 15.71	46.4 ± 11.05	9 (3.55–14.45)	0.001^c^
RASS score at T2				
> 0 agitated range, no. (%)	8 (16.33%)	1 (2.04%)	0.09 (0.01–0.79)	0.012^b^
0 alert and calm, no. (%)	26 (53.06%)	35 (71.43%)	2.21 (0.96–5.10)	0.095^b^
< 0 sedation range, no. (%)	15 (30.61%)	13 (26.53%)	0.64 (0.26–1.58)	0.367^b^
CPOT score at T2, median (25% – 75%)	1.04 ± 1.74	0.33 ± 1.14	0.71(0.12–1.31)	0.003^a^
Hypoxemia occurrence at T3, no. (%)	28 (57.14%)	7(14.29%)	8 (3.00–21.32)	< 0.001^b^
Hypoxemia occurrence at T4, no. (%)	6 (12.24%)	1 (2.04%)	0.15 (0.02–1.29)	0.111^b^
Grade of sedation at T3 (Grade 2/Grade 3), no. (%)	13(27%)/36(73%)	13(27%)/36(73%)	1 (0.41–2.45)	1^b^
Postoperative pain at T4, no. (%)	1 (2.04%)	1 (2.04%)	1 (0.61–16.45)	1^b^
Residual sedation at T4, no. (%)	0 (0%)	0 (0%)		
Nausea and vomiting at T4, no. (%)	4 (8.16%)	1 (2.04%)	0.23 (0.03–2.18)	0.362^b^
Pharyngalgia at T4, no. (%)	15 (30.61%)	7 (14.29%)	0.38 (0.14–1.03)	0.89^b^

*Abbreviations: PACU* Post-anaesthesia care unit, *RASS* Richmond Agitation-Sedation Scale, *CPOT* Critical-Care Pain Observation Tool, *CI* Confidence interval, *R* TCI of remifentanil, *L* Topical lidocaine via the perforated outer cuff, *CI* Confidence interval, *OR* Odds ratio, *SD* Standard deviation, *T0* One day before surgery, *T2* 1 min before tracheal extubation, *T3* 5 min after tracheal extubation, *T4* 20 min after tracheal extubation. Elevation of blood pressure, increase by 30% from the mean arterial pressure at T0; elevation of heart rate, increase by 30% from the heart rate at T0; hypoxemia, Spo_2_ less than 95%; grade of sedation: Grade 0: deeply sedated and unresponsive; Grade 1: sedated but responsive to light glabellar tap; Grade 2: sedated but responsive to normal voice; Grade 3: awake and responsive. Postoperative pain, more than 5 points on the visual analogue scale (VAS); residual sedation, less than Grade 2; nausea and vomiting, need for drugs; pharyngalgia, swallowing with more than 3 points on the VAS

^a^ Mann–Whitney U test

^b^ Fisher’s exact test

^c^ *t test*

## Discussion

Compared with remifentanil, lidocaine via the perforated outer cuff more effectively decreased the incidence of cough during recovery, the rate of heart rate elevation, the spontaneous respiration recovery time, the extubation time, the duration of PACU stay, RASS scores in the agitated range, CPOT scores, and hypoxemia at T3. The other recovery parameters did not differ between the two groups.

A meta-analysis comparing treatments for cough suppression during recovery from general anaesthesia revealed that topical lidocaine is the least effective compared to dexmedetomidine, remifentanil, and fentanyl [19]. In addition, the extubation time was delayed after lidocaine administration via the intracuff compared with remifentanil [19]. However, the results of this study were not consistent with the ranking of treatments suppressing cough. Lidocaine administered via a topical route had a better antitussive effect and led to a shortened extubation time compared to remifentanil. The method of topical lidocaine administration in this study was different from the method of tracheal topical lidocaine used at present in the clinic. Topical tracheal lidocaine is currently administered via the tracheal tube or intracuff, by topical administration to the glottis or onto the tracheal mucosa via both upper and lower endotracheal tube cuffs [19]. The reduced efficacy observed for lidocaine administration in the clinic compared to that in this study is possibly due to the following reasons. Lidocaine administered via the tracheal tube before intubation for surgeries lasting < 2 h is undesirable for suppressing cough during recovery [20]. However, the duration of general anaesthesia in this study was > 2 h. The cough-suppressing effect of alkalinised lidocaine intracuff is dependent on the time necessary for the alkalinised lidocaine to permeate across the cuff membrane [21]. A dual-cuff ETT avoids this issue, as the perforations in the outer cuff allow lidocaine to rapidly distribute around the dual-cuff ETT. Lidocaine administered via a laryngotracheal instillation of topical anaesthesia (LITA) tube is distributed onto the glottis or both the upper and lower cuff before extubation [22, 13], resulting in cough suppression in only 75% [13] of patients during recovery, which is a lower rate than that obtained in this study. The tube cuff is a mechanical barrier that hinders the distribution of topical lidocaine anaesthesia to the tracheal mucosa [13]. The outer cuff of the modified dual-cuff ETT can break the mechanical barrier and does not require deflation for the administration of topical lidocaine onto the tracheal mucosa. The tracheal mucosa is under topical anaesthesia with lidocaine via the perforated outer cuff of the ETT, which reduces the stimulation of the tracheal mucosa. These factors could potentially explain the lower RASS and CPOT scores recorded in Group L in this study.

The high efficiency of lidocaine compared with remifentanil in this study was possibly due to the use of topical anaesthesia. The two methods of administration had different antitussive effects because of the primary sites of action. Remifentanil (a μ-opioid receptor activator) indirectly elicits cough-suppressive effects via opioids, which act to suppress sensory nerve activity [23, 24] because μ-opioid receptor activators inhibit calcium currents [25]; however, the stimulation of the tracheal mucosa in contact with the cuff is not reduced. The cough-suppressive effect of lidocaine topical anaesthesia is peripherally mediated, not centrally mediated, because the plasma concentrations (peak serum levels < 1.63 μg/mL) of lidocaine topical anaesthesia do not reach the level needed for cough-suppressive effects (> 3 μg/mL) [13, 20, 26]. The method of lidocaine administration in this study, which used a topical approach, could possibly explain the high efficiency of lidocaine compared with remifentanil. In this study, topical lidocaine adequately and directly reduced the stimulation of the tracheal mucosa in contact with the cuff without restricting the timing of administration or deflating the internal cuff.

In addition, lidocaine resulted in better recovery after respiratory complications and a shorter duration of PACU stay. In contrast, remifentanil increased the occurrence of hypoxemia (57.14%) at T3, the spontaneous respiration recovery time, and the extubation time. This is consistent with Beloeil's study [27], which reported hypoxemia in 61% of the patients treated with remifentanil. Compared with remifentanil, the lower respiratory complications observed in patients treated with lidocaine in this study could be because they were remifentanil-free during recovery from general anaesthesia.

Compared to remifentanil, intravenous (IV) lidocaine increased residual sedation [16]. The lidocaine serum concentration was greater than 3 μg/ml with the administration of 2 mg/kg IV lidocaine [28]. However, a 2 mg/kg dose of 4% lidocaine via topical distribution was used by Diachun [13] before extubation, which resulted in peak serum levels < 1.633 μg/ml. The lidocaine doses in this study were lower than those used in Lee et al.'s study [16], which may explain why the lidocaine used in this study did not increase residual sedation relative to TCI remifentanil.

### Limitations and generalisability

This study had several limitations. First, only women were included in the study because women have a higher incidence of thyroid cancer [29], and the incidence of cough shows significant sex differences [30]. Second, an internal cuff carries the risk of air leakage. Thus, the internal cuff must be checked for leakage prior to intubation. Third, the injection of lidocaine, saline solution, or 20 ml of air via the outer cuff could induce cough. A sufficient anaesthesia depth minimises the risk of effect-site TCI remifentanil (2.0 ng/ml) and sevoflurane (1.4–1.5%). Fourth, NIM tubes are fairly standard practice elsewhere, which may make this new modified dual-cuff ETT less useful in clinical practice for certain patients undergoing thyroidectomy. However, cough during recovery was chosen as the primary outcome for further study of the potential benefits of the dual-cuff ETT, such as reducing airborne material and droplets during the recovery of patients with suspected or confirmed COVID-19 [7] or other cough-related adverse events.

## Conclusion

In conclusion, lidocaine administered via the perforated outer cuff of the ETT significantly improved recovery from general anaesthesia compared with remifentanil in female patients after thyroidectomy.

## Supplementary Information

**Additional file 1: Supplemental Figure 1.** DISPOSABLE REINFORCED TRACHEAL INTUBATION DOUBLE BALLOON DOSING-TYPE. A dual-cuff ETT contains a white internal cuff covered by a blue outer cuff. Two small-bore channels incorporated within the wall of the endotracheal tube are the channels that deliver air to the cuffs.

**Additional file 2: Supplemental Figure 2.** Dyed areas (red areas) with different dye liquid volumes and numbers of holes (A). The experiment was performed in triplicate for each combination. Graph paper (25 mm^2^ per unit) represents the area of the simulative trachea in contact with the perforated outer cuff of the dual-cuff endotracheal tube. The optimal scheme to achieve the even distribution of dye liquid around the endotracheal tube cuff was 16 holes and 4 ml of dye liquid. ^#^*P *< 0.05 *vs.* 4 ml of dye liquid volume. ^&^*P *< 0.05 *vs.* 8 holes. The structure of a modified dual-cuff endotracheal tube (B). This modified dual-cuff endotracheal tube contains an internal cuff covered by an outer cuff. The function of the internal cuff (black cuff) is the same as that of the single cuff in traditional endotracheal tubes. The small-bore channel incorporated within the wall of the endotracheal tube is the channel that delivers lidocaine to the outer cuff (green area). The outer cuff contains 16 small holes (each hole was 1 mm in diameter), which are evenly arranged in two rows (8 holes per row). The first row of holes (red area) and the second row of holes (blue area) are arranged at one-third and two-thirds of the outer cuff contacted internal cuff (the number represents the distribution of small holes in the outer cuff), respectively. Topical lidocaine is administered via small holes in the perforated outer cuff.

**Additional file 3: Supplemental Video 1.** The process of the perforated outer cuff.

**Additional file 4: Supplemental Video 2.** One patient was intubated with the dual-cuff ETT while sitting up in bed and signing her name.

**Additional file 5: Supplemental Video 3.** Lidocaine pass through the perforated outer cuff.

## Data Availability

The data used and analysed in this study are available from the corresponding author.

## Abbreviations

ETT: Endotracheal tube
OD: Odds ratio
CI: Confidence interval
COVID-19: Coronavirus disease
TCI: Target-controlled infusion
ASA: American Society of Anesthesiologists
R: Remifentanil
L: Lidocaine
Ce: Effect-site concentration
PACU: Post-anaesthesia care unit
MAP: Mean arterial pressure
HR: Heart rate
ETCO_2_: End-tidal carbon dioxide
RASS: Richmond Agitation-Sedation Scale
CPOT: Critical-Care Pain Observation Tool
SGS: Sedation Grading System
LITA: Laryngotracheal instillation of topical anaesthesia
IV: Intravenous
