# Supplementary figures and images for: Effects of lidocaine administration via the perforated outer cuff of a dual-cuff endotracheal tube and remifentanil administration on recovery from general anaesthesia for female patients undergoing thyroidectomy: a single centre, double-blind, randomised study
Source: BMC Anesthesiol. 2022 Jun 22;22:194. doi: 10.1186/s12871-022-01734-1 (PMC9213641; doi:10.1186/s12871-022-01734-1)

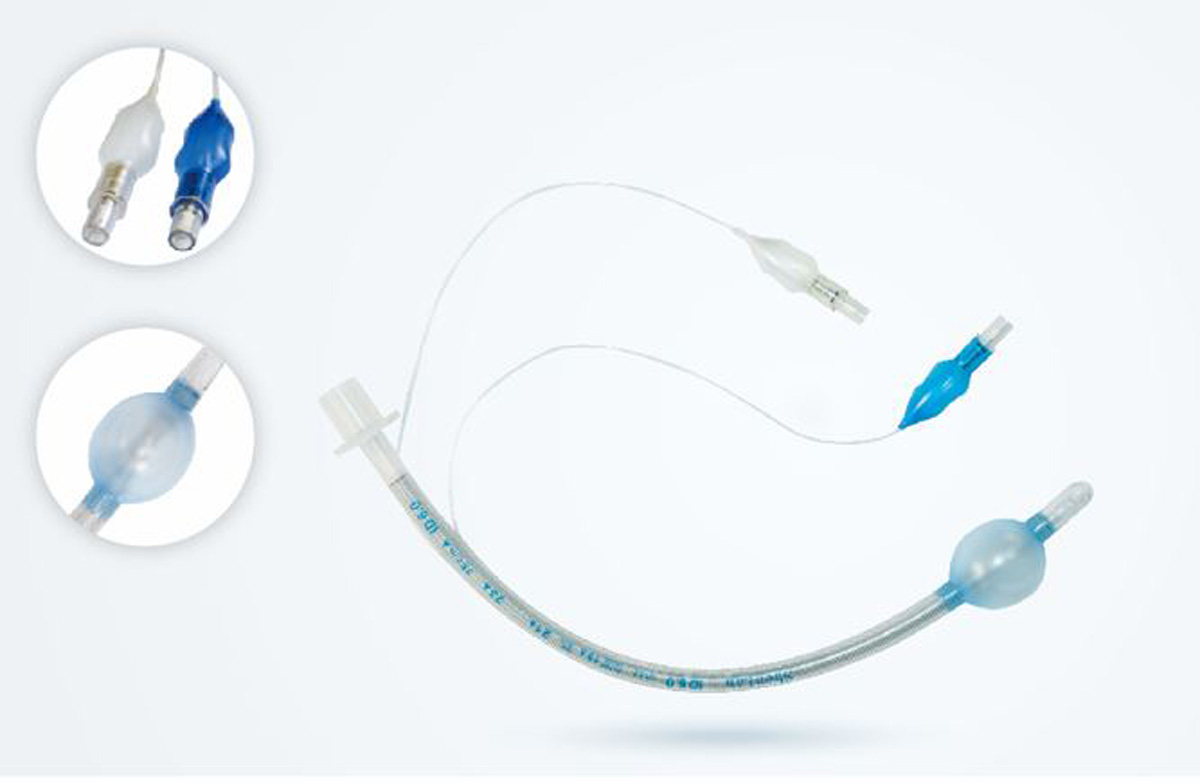

Supplement: Supplementary file 1 — Additional file 1: Supplemental Figure 1. DISPOSABLE REINFORCED TRACHEAL INTUBATION DOUBLE BALLOON DOSING-TYPE. A dual-cuff ETT contains a white internal cuff covered by a blue outer cuff. Two small-bore channels incorporated within the wall of the endotracheal tube are the channels that deliver air to the cuffs. [file 12871_2022_1734_MOESM1_ESM.jpg]

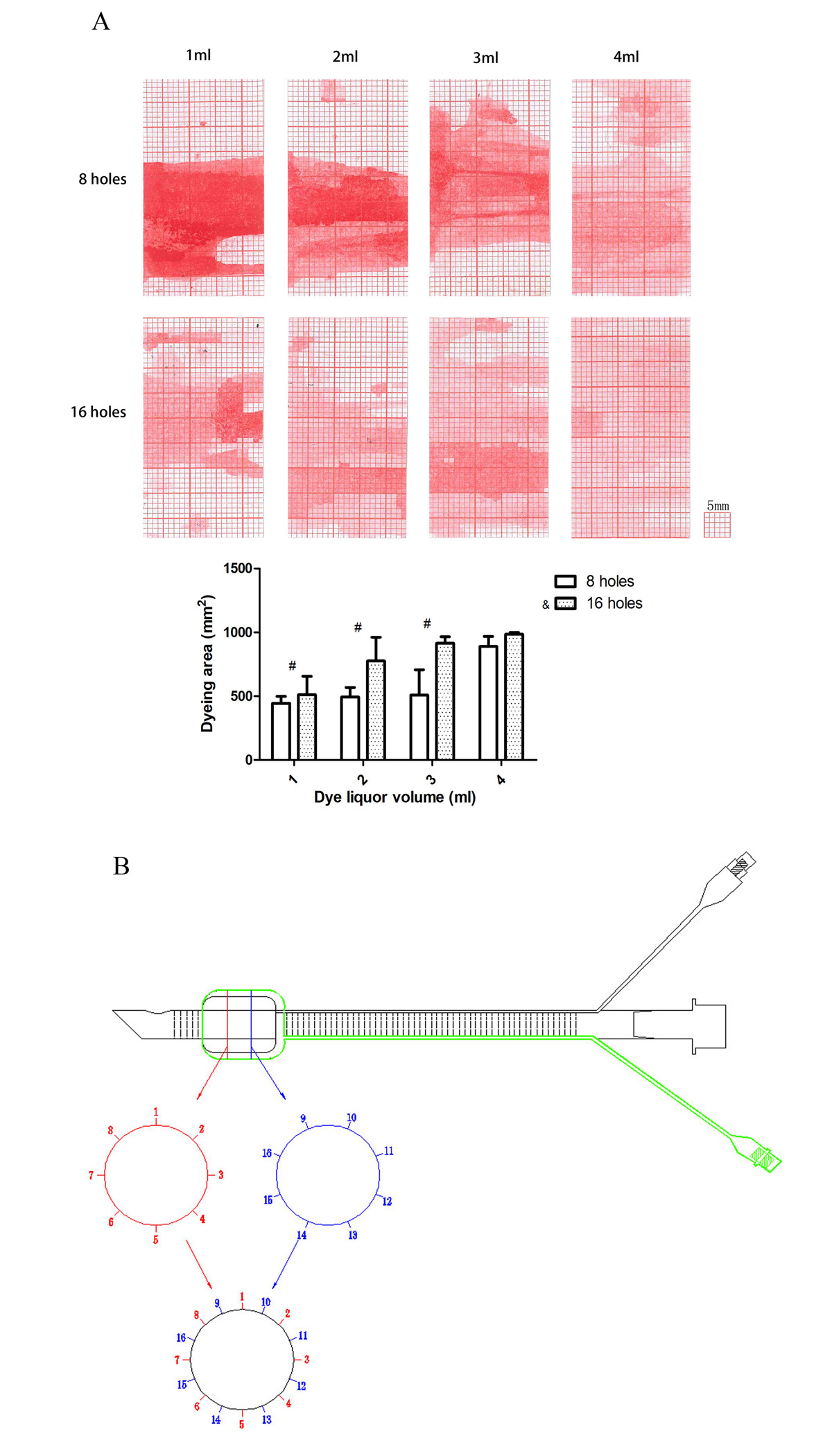

Supplement: Supplementary file 2 — Additional file 2: Supplemental Figure 2. Dyed areas (red areas) with different dye liquid volumes and numbers of holes (A). The experiment was performed in triplicate for each combination. Graph paper (25 mm2 per unit) represents the area of the simulative trachea in contact with the perforated outer cuff of the dual-cuff endotracheal tube. The optimal scheme to achieve the even distribution of dye liquid around the endotracheal tube cuff was 16 holes and 4 ml of dye liquid. #P < 0.05 vs. 4 ml of dye liquid volume. &P < 0.05 vs. 8 holes. The structure of a modified dual-cuff endotracheal tube (B). This modified dual-cuff endotracheal tube contains an internal cuff covered by an outer cuff. The function of the internal cuff (black cuff) is the same as that of the single cuff in traditional endotracheal tubes. The small-bore channel incorporated within the wall of the endotracheal tube is the channel that delivers lidocaine to the outer cuff (green area). The outer cuff contains 16 small holes (each hole was 1 mm in diameter), which are evenly arranged in two rows (8 holes per row). The first row of holes (red area) and the second row of holes (blue area) are arranged at one-third and two-thirds of the outer cuff contacted internal cuff (the number represents the distribution of small holes in the outer cuff), respectively. Topical lidocaine is administered via small holes in the perforated outer cuff. [file 12871_2022_1734_MOESM2_ESM.jpg]
